# Enhancing Engagement with Stop Smoking Services among Lower Socioeconomic Groups across the UK: A Qualitative Study using the Behaviour Change Wheel

**DOI:** 10.1093/ntr/ntaf256

**Published:** 2025-12-19

**Authors:** Pamela Smith, Lucia Dahlby, Evgeniya Plotnikova, Rebecca-Bell Williams, Rebecca Thorley, Rachael Murray, Fiona Dobbie, Tessa Langley, Ilze Bogdanovica, Kate Brain, Leah Jayes

**Affiliations:** Division of Population Medicine, Cardiff University School of Medicine, Cardiff CF14 4YS, UK; Usher Institute, University of Edinburgh, Edinburgh EH16, UK; Usher Institute, University of Edinburgh, Edinburgh EH16, UK; Faculty of Medicine and Health Sciences, University of Nottingham, Nottingham NG5 1PB, UK; Faculty of Medicine and Health Sciences, University of Nottingham, Nottingham NG5 1PB, UK; Faculty of Medicine and Health Sciences, University of Nottingham, Nottingham NG5 1PB, UK; Usher Institute, University of Edinburgh, Edinburgh EH16, UK; Faculty of Medicine and Health Sciences, University of Nottingham, Nottingham NG5 1PB, UK; Faculty of Medicine and Health Sciences, University of Nottingham, Nottingham NG5 1PB, UK; Division of Population Medicine, Cardiff University School of Medicine, Cardiff CF14 4YS, UK; School of Social Sciences, Nottingham Trent University, Nottingham NG1 4BU, UK

## Abstract

**Introduction:**

Individuals who use a stop smoking service (SSS) in the UK are three times more likely to quit. Uptake of SSS is limited among lower socioeconomic (LSE) groups and efforts are needed to understand how to improve the appeal, acceptability, and accessibility of SSS.

**Methods:**

Semi-structured interviews with 114 participants from the four UK devolved nations who had a current or recent smoking history and who had previously accessed or may potentially access a SSS. Participants were recruited via Facebook, third sector organizations, and a market research company. Telephone-based interviews were analyzed using thematic analysis. The Behaviour Change Wheel was used to develop the interview topic guide, inform thematic analysis, and identify strategies to increase engagement with SSS.

**Results:**

Barriers included low awareness and understanding regarding the availability and content of SSS, a lack of free time to access SSS and negative beliefs regarding the efficacy of SSS support. Facilitators included more frequent and continued flexible support delivered using a range of modes, access to free Nicotine Replacement Therapy (NRT), and rapport with the advisor. Intervention functions were identified to address these barriers and facilitators via educational efforts to raise awareness of SSS, environmental restructuring to provide a flexible approach to delivery, and community champions to encourage and enable SSS engagement in the target population.

**Conclusions:**

Providing further community-based efforts along with a more holistic approach to delivering behavioural support and NRT has strong potential to maximize SSS reach, engagement, and acceptability.

**Implications:**

The current study provides insights into the barriers and facilitators to engaging with and accessing SSS among LSE groups and indicates that interventions to increase capability, motivation, and opportunity are required. Recommendations from this work for tailoring UK SSS for LSE groups can be used to inform future tobacco control policy including engagement strategies at a local and national level.

## Introduction

Smoking is still the leading cause of global preventable deaths killing more than eight million people each year.^1^ In 2023, around six million UK adults smoked and although smoking prevalence has declined, prevalence among lower socioeconomic (LSE) groups remains high.^2^^,^^3^ Stop smoking services (SSS) were initially set up in England in 1999 to target areas of high deprivation and were later rolled out to other parts of the UK in 2000.^4^ Although SSS are commissioned differently across the four devolved nations, they largely offer the same support, a combination of behavioural and pharmacological support (either dual nicotine replacement therapy products or Varenicline, Bupropion, Cytisine [if available]) for around 12 weeks via one-to-one support (face-to-face, telephone/virtual, or web-based apps). Currently only some SSS in England offer e-cigarettes as a cessation aid. Previous work has demonstrated that such gold standard smoking cessation support (nicotine replacement therapy combined with behavioural counselling) is the most effective way to stop smoking^5^ and individuals who use SSS are three times more likely to stop smoking than those who try to quit without support.^6^

Existing research demonstrates that there are a variety of factors that contribute to smoking cessation rates being low for more disadvantaged smokers including age, amount of friends and family who smoke and nicotine dependence.^7^^,^^8^ SSS have made important contributions to the reduction of smoking prevalence and although services employ techniques, such as targeted advertisements, to specifically target LSE groups, uptake is still not equal across different socioeconomic groups and individuals from LSE backgrounds are still less likely to engage with a SSS.^9-11^ Previous literature suggests that behavioural smoking cessation interventions that are tailored to and delivered at the individual level may prove more successful compared to population level interventions.^12^^,^^13^ Additionally, smoking cessation interventions are more likely to be effective if they are informed by a theoretical understanding of how smoking cessation interacts with smoking-related beliefs and behaviours in LSE groups.^14^ Further understanding of reasons why the uptake of SSS is particularly low among LSE groups is needed to modify aspects of SSS to overcome barriers to accessing and engaging with SSS. We sought to develop recommendations for tailoring SSS to increase engagement in this population guided by the COM-B model and theoretical domains framework (TDF) of the Behaviour Change Wheel.^15^^,^^16^

**Figure 1:**
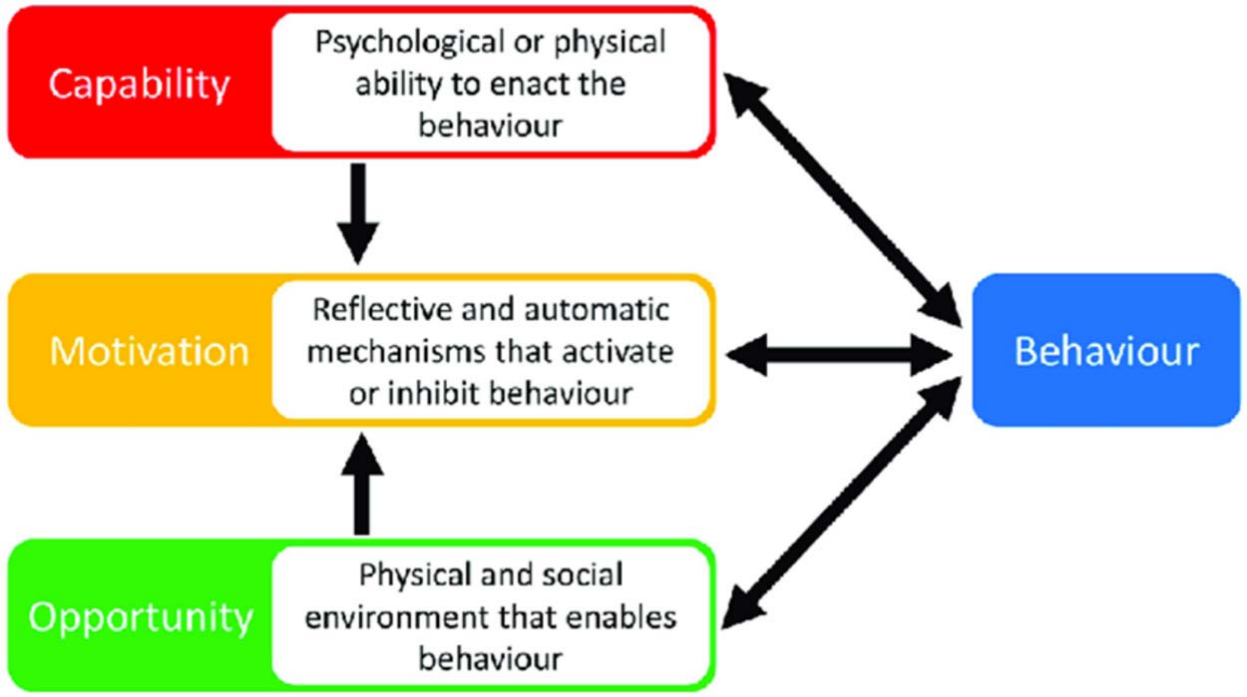
COM-B model.

COM-B describes how Capability, Opportunity, and Motivation are components needed in order for Behaviour to change.^15^ Capability can be psychological or physical, Opportunity can be social or physical, and Motivation can be automatic or reflective (see Figure 1^16^). In turn, the TDF provides a more granular lens through which to understand Capability (eg, knowledge, physical skills), Opportunity (social influences, environmental context, and resources), and Motivation (eg, emotions, beliefs about capabilities).^17^^,^^18^ The COM-B model and TDF are used together to identify what needs to change in order to bring about the target behaviour (engaging with SSS). Finally, intervention functions can be considered; these functions are broad categories that align to the COM-B model through which an intervention can change behaviour (eg, an intervention function linked to Opportunity is “education”) (Table S1).

The aim of this study was to explore the appeal, acceptability, and accessibility of UK SSS for people in LSE groups and to understand the barriers and facilitators to engagement, as well as areas of opportunity for service providers. Specific objectives were to gather evidence from qualitative interviews to identify barriers and facilitators to accessing and engaging with SSS and identify the type of intervention functions that are required to increase engagement with and access to SSS.

## Materials and Methods

### Design

Qualitative semi-structured interviews were conducted with previous and potential SSS users from LSE groups across England, Wales, Scotland, and Northern Ireland. Interviews explored the type of support SSS offered, barriers and facilitators to accessing a SSS, and suggestions for potential recommendations to improve access to SSS. The interview schedule was guided by the COM-B model as a framework to enable understanding of Capability, Opportunity, and Motivational barriers and facilitators to SSS engagement (Behaviour).

### Procedure

Ethical approval was gained from the University of Nottingham, Faculty of Medicine and Health Sciences Research ethics committee (ref: FMHS 250-0423). Participants were recruited through third sector organizations (eg, charities and local community groups), paid targeted Facebook adverts, and by a specialist market research company (Taylor McKenzie Research). Potential participants were screened based on eligibility criteria which included^1^: smoking status (smoked at least one cigarette in the last 4 weeks, smoked in the past but now uses an e-cigarette or quit smoking within the last 12 months)^2^; meeting at least two out of three individual indicators of deprivation (education, living arrangement, and household income)^3^; and postcode that demonstrates residency in the UK. Facebook adverts were live for three months (July–October 2023) and reposted several times each month.

Interviews were audio-recorded and transcribed verbatim. The topic guide was developed so that similar areas of interest were covered with each participant but also allowed flexibility to enable issues that were important to be discussed. Interviews lasted between 35 and 45 minutes.

### Participants

Convenience and purposeful sampling methods were combined to recruit participants from LSE groups who had previously accessed SSS or may potentially access a SSS in the future. Sampling size was determined by satisfying the following criteria: (1) provisional sample sizes outlined in the funders commissioning brief (eg, a request to sample more service users in England due to its population relative to the other devolved nations and the variability in service commission, management and delivery in England compared to other nations), (2) sample diversity required for qualitative research (eg, those who had and had not accessed a SSS before), and (3) achieving sufficiently rich data.^19^ Sampling quotas were applied to obtain a mixture of individuals from across the devolved UK nations, with representation by gender, ethnicity, age, smoking status, and access of SSS.

### Analysis

Interview data were analyzed using the COM-B model and TDF as a framework for identifying key mechanisms of action for engaging with SSS (the target behaviour). Firstly, transcripts were analyzed thematically^20^ and supported by qualitative data analysis software (NVivo 12). P.S., L.J., R.T., L.D., E.P., and R.B.W. all coded transcripts independently and then met to discuss disparate views by comparing identified themes and codes within transcripts as well as across the dataset. Twenty percent of transcripts were double coded by at least two members of the research team and discrepancies were noted and resolved through discussions. Codes from the thematic analysis were then mapped deductively on to COM-B and TDF constructs, enabling the identification of the key barriers and facilitators to SSS engagement among LSE groups. All codes were mapped onto at least one of the domains.

The next step in the analysis process involved identifying the types of intervention likely to encourage engagement with SSS in LSE groups. The previous step of identifying relevant COM-B and TDF domains was then linked to functions through which an intervention can change behaviour. The nine intervention functions and their related COM-B components along with TDF domains identified in step 1 were considered using the intervention function matrix. For example, barriers and facilitators that were coded under “psychological capability” may require intervention functions related to “education” or “persuasion.” The affordability, practicability, effectiveness/cost-effectiveness, acceptability, side effects/safety, equity (APEASE) criteria was used to guide judgment for selecting intervention functions that were most appropriate.^16^ P.S. and L.J. both assessed these separately and then met to discuss and resolve any discrepancies.

## Results

### Sample Characteristics

We conducted 114 interviews with a range of participants (see Table S2) from England (*n* = 40), Scotland (*n* = 24), Wales (*n* = 25), and Northern Ireland (*n* = 25). Our sample had an even split of males and females and with nearly two thirds of these reporting that they were current smokers. Just over two thirds of the participants (*n* = 80) had ever accessed a SSS with almost half of those accessing one within the last year (*n* = 36). Interviewees mainly consisted of White British (*n* = 94), lived in an urban environment (*n* = 82), and were under 60 years of age (*n* = 97). Participants predominantly lived in rental accommodation (*n* = 60), had left school with minimal qualifications (GCSE or below) (*n* = 77), and were from the most deprived quintile (*n* = 57) (Table S2).

### Mapping the Identified Codes from Thematic Analysis onto COM-B and TDF Domains

#### Barriers and Facilitators to Accessing and Engaging with SSS

The following findings relate to perceived barriers and facilitators that were identified as themes during the thematic analysis. These findings were gathered from those who had never accessed a SSS alongside the first-hand experiences of those who have and are discussed in relation to the COM-B components and TDF domains (see Table S3).

### Psychological Capability

#### Knowledge

A range of barriers related to knowledge surrounding SSS was discussed. The majority of participants who had never accessed a SSS lacked knowledge that service to support them to stop smoking existed. For those who did have some awareness of SSS, there was limited understanding of what support the services offered. For example, participants were often unaware that they could access free NRT products and that behavioural support could be delivered over the telephone. Many participants perceived SSS as being an outdated approach to smoking cessation and felt that the service was aimed at an “older generation.” In regard to mode of delivery, the majority of participants were also unaware that there are a range of settings (community, pharmacy, GP) in which they could receive stop smoking support. Additionally, a lack of understanding of the use of e-cigarettes to support smoking cessation was discussed. Participants generally felt that they did not possess enough knowledge on the use of e-cigarettes and suggested the need for more guidance on their use as a cessation tool.

### Physical Opportunity

#### Environmental Context and Resources

Generally, participants who had previously accessed a SSS felt there was a lack of flexibility in mode of delivery, for example only having telephone-based support available, and viewed this as a barrier in the potential future use of a SSS. Mode of delivery of SSS varies across the UK and there were mixed views regarding the optimal mode; some participants valued over-the-telephone support as they could fit this around a busy schedule, whereas others felt that in-person behavioural support was preferable as this more easily allowed for rapport building.

Another barrier to accessing SSS was the frequency and continuity of behavioural support. Many participants mentioned the need for more contact from an advisor in between the scheduled weekly sessions and felt support beyond the standard SSS 12-week program would be beneficial. They also discussed that they would be more likely to use a SSS again if the NRT support was provided beyond the current standard 12-week program. Some participants suggested they would have valued being able to contact an advisor in-between their weekly sessions through, for example, WhatsApp or text messaging, which is currently not formally offered within SSS.

Having community-based support in SSS was a facilitator for access among the majority of participants. Although participants acknowledged the need for remote service delivery for those who are unable to attend in-person support, many believed that having easy-to-access community-based support would influence them in accessing a SSS in the future. Participants also felt that there was a lack of choice regarding the location of in-person behavioural support and whether one-to-one or group support was available. Rapport building with an advisor was also viewed as an important facilitator for both participants who had previously accessed SSS and those who had not.

Those who had previously accessed a SSS through a local pharmacy felt that they would have liked to have received more behavioural support in addition to NRT. Free NRT was frequently discussed by participants as a useful component of a SSS and one that enables them to acquire products, which they would likely not be able to purchase themselves due to cost. However, some participants had experienced delays in accessing NRT through SSS.

### Social Opportunity

#### Social Influences

Participants discussed the importance of social networks in supporting them during a quit attempt and many reflected on having experienced a lack of positive social support in relation to smoking cessation. The concept of a peer support model in SSS was discussed as a potential facilitator for accessing and engaging with a SSS as participants felt that this would enable encouragement and learning from others who have successfully quit smoking. Additionally, participants felt that the use of key community members to enable dissemination of information about SSS might help to improve awareness and understanding.

All participants discussed stigma related to smoking, specifically the fear of failing when making a quit attempt and therefore wanting to quit on their own, as this would come without judgment from others. This meant that participants did not want to quit through the support of a SSS and would prefer to keep their quit journey private. For those who had previously accessed a SSS or would consider using a SSS in the future, the use of an advisor who had a smoking history was desirable. Participants also felt that having an advisor who is compassionate and empathetic would be valuable when trying to stop smoking.

### Motivation

#### Beliefs about Capabilities and Consequences

The majority of participants who had never accessed a SSS described negative beliefs about the behavioural support that SSS offer. When prompted to discuss why they felt this way, many participants discussed that meeting people, whether in a group setting or one-to-one, and talking about smoking would not motivate them to quit. Many participants felt that they would be uncomfortable opening up to others about their smoking behaviour. In relation to negative beliefs about NRT efficacy, some participants reflected on having previously used NRT during a quit attempt and experiencing side effects that have put them off using them. Participants also reported hearing negative stories from friends and/or family who have utilised NRT previously. Similarly, some participants held negative beliefs regarding the safety and efficacy of e-cigarettes with many feeling uncertain as to whether they would utilise them as a cessation tool during a future quit attempt.

### Linking COM-B and TDF Domains to Intervention Functions

#### Types of Intervention Likely to Encourage Engagement with SSS

The process of mapping COM-B components onto the intervention function matrix focuses on seven intervention functions needed to be considered. These functions included: *education, persuasion, incentivisation, training, environmental restructuring, modeling*, and *enablement* (Table 1). After applying the APEASE criteria, all seven functions were identified as eligible for inclusion.

**Table 1:** Intervention Function Matrix

COM-B components	Intervention functions								
	Education	Persuasion	Incentivisation	Coercion	Training	Restriction	Environmental restructuring	Modeling	Enablement
Psychological capability									
Physical opportunity									
Social opportunity									
Reflective motivation									

As illustrated in Table 1, *Enablement* was included as an intervention function because reducing barriers to SSS in the UK is important for engagement with services from the target population to be achieved. *Education* formed a central part of the intervention, involving an increase in the provision of information as a mechanism to improve awareness of SSS and knowledge of what support these services involve, such as free pharmacotherapy and e-cigarettes. *Persuasion* was selected to demonstrate the potential to encourage and advice individuals who want to stop smoking, or may want to stop smoking in the future, to engage with a SSS. *Training* was selected as an intervention function to reflect that engagement with SSS could be achieved via training SSS practitioners to confidently discuss e-cigarettes and provide guidance around usage. *Environmental restructuring* was also selected as it is suggested that the social and physical context of individuals should be adjusted to encourage engagement with SSS. For example, having more community-based SSS and making behavioural support more accessible. *Incentivisation* was included to provide feedback on behaviour (ie, smoking cessation) for those who are engaging with a SSS. Finally, *modeling* was selected as a function due to the importance of social support and networks for individuals from LSE groups.

## Discussion

The behaviour change wheel (BCW) was applied as a mechanism to develop recommendations for enhancing existing SSS in the UK for LSE groups. To our knowledge, the current study is the first to apply the BCW to develop recommendations for improving engagement with SSS among individuals from LSE groups. Through implementing this systematic approach, we have been able to provide insights into the barriers and facilitators to engaging with and accessing SSS among LSE groups. We used the COM-B and TDF^15^ to map key barriers and facilitators, which then helped to shape the theoretical basis for developing recommendations to enhance SSS in the UK for LSE groups.

Evidence from our study indicates that interventions to increase capability, motivation, and opportunity are needed to improve engagement with SSS among LSE groups in the UK. Similarly to previous work,^21^^,^^22^ individuals lacked knowledge surrounding what SSS are available to them, how to access, and what these services offer. Our research findings suggest that to increase the psychological capability of potential SSS users efforts should be made to educate LSE groups about SSS via local, targeted promotion. Previous mass media campaigns have focused on changing smoking behaviour and encouraging quitting,^23^^,^^24^ with a lack of evidence of their effectiveness and reach to individuals in LSE groups. An increase in targeted advertisement of local SSS would be beneficial in improving awareness and understanding of what services exist and what they offer. The use of education through advertisement to demonstrate how service provision has evolved over the last 30 years may help to persuade those who feel SSS are “outdated.” Further efforts to help raise awareness of what SSS involve (eg, access to free NRT, a range of modes of delivery for behavioural support) may encourage those who are looking to quit smoking to engage with services. For example, individual-level along with service-level persuasion surrounding the use of pharmacological support would also help to encourage potential and current SSS users to engage with and access this form of support.

Enhancing social influences in SSS by advisors and trusted individuals encouraging others to quit with the support of a service and not on their own may improve engagement with behavioural support. The use of a credible source such as an existing community group to present information regarding access SSS may help to restructure the social environment through one-on-one discussions about local SSS and how to access support if they are trying to stop smoking. One study completed across three Local Authorities in England found that outreach activities that are delivered through effective community partnerships can raise awareness of SSS, remove access barriers, and generate referral.^25^ Other social support strategies could involve emphasizing facilitators to engaging and accessing SSS, such as free, easy-to-access NRT support. SSS should further implement a range of modes for behavioural support to be received for LSE groups.^26^

Further development and strengthening collaboration with third sector organisations, community services, and local authorities was a suggested intervention strategy to increase visibility, access, and uptake among LSE group. For example, community partners could be encouraged to raise awareness of local SSS and gain training in the delivery of Very Brief Advice for smoking cessation through a Making Every Contact Count approach in which partners can signpost individuals from LSE groups to local SSS.^27^ This recommendation is an example of understanding the needs of LSE groups in relation to smoking cessation and demonstrates the importance of continued efforts to embed mechanisms for improving awareness of and engagement with SSS in the local needs and preferences of this population.

Similarly, to previous research on SSS, this study provides evidence showing the importance of providing flexible, tailored stop smoking support to LSE groups as a means to help address health inequalities and attempt to meet the needs of this population.^13^^,^^28^^,^^29^ For example, participants felt that behavioural support would not be beneficial to them when trying to quit, and they would prefer to quit alone, without talking to someone. In the case of this finding, we identified that reflective motivation should be addressed via efforts to reframe negative beliefs held by potential service users around behavioural support as part of the package that SSS offer. Our work demonstrates that SSS should address boosting an individual’s confidence in their ability to quit with others, particularly with those who are likely to be less engaged with the behavioural support aspect of SSS.

Our findings demonstrate the importance of enhancing behavioural support to include further emphasis on the effectiveness of NRT as well as including any potential side effects. Furthermore, many participants felt that there is a lack of support from SSS regarding the use of e-cigarettes as a cessation tool, particularly in Wales, Scotland, and Northern Ireland where SSS do not currently provide e-cigarettes. There were mixed findings in relation to the use of e-cigarettes, with some participants mentioning wanting this form of support and others viewing e-cigarettes as potentially harmful. Despite the UK government supporting the use of e-cigarettes for smoking cessation, harm perception of e-cigarettes has worsened among those who smoke over the last 10 years^30^ and findings from this study demonstrate the need to consider this when tailoring support for LSE groups. Behavioural support for LSE groups should include clear evidence-based guidance surrounding vaping and smoking cessation advisors should aim to engage in supporting discussions regarding the use of e-cigarettes.^31^ Interestingly, there were very few participants who spoke about the use of smoking cessation medications (Varenicline, Bupropion, Cytisine) to support a quit attempt. This is likely a reflection of changes in their prescription licensing over recent years across the UK (both Varenicline and Bupropion have been withdrawn in recent years and Cytisine has only been available since early 2024). More research exploring barriers or facilitators to access these types of medications for those in LSE groups would be warranted.

In relation to e-cigarettes, further training for SSS providers surrounding discussions about e-cigarettes would enable service users to feel supported when it comes to conversations around e-cigarettes as a cessation tool. Incentivisation through the use of certificates to recognize and praise specific, personalized milestones in a person’s quit journey (eg, 2 weeks quit, went on holiday and did not smoke) may help to increase morale and reward for those using a SSS.

Participants expressed a preference for having more community-based, in-person support available along with offering more flexible and continued support past 12 weeks of support that is currently offered in SSS. Restructuring the physical environment means changing the physical environment to aid in facilitating the performance of a behaviour.^16^ Our research demonstrates that barriers to engaging with SSS may be reduced by offering more community-based services with a longer treatment program length to support LSE groups to stop smoking. Alternatively, we found that some individuals may prefer SSS delivery to include remote/online options. These findings further demonstrates the importance of offering a flexible, person-centered approach to the delivery of smoking cessation support.

### Strengths and Limitations

Our research was underpinned by a strong theoretical base, which was applied to help identify barriers and facilitators. We adopted a systematic approach using the BCW, which ensures that the research team considered the target audience at each stage of the development. A limitation of using the BCW is the subjectivity in the process of developing interventions; however, this was minimized by having two team members involved in the mapping process.

We recruited a large sample of participants with varied demographic characteristics. Furthermore, the use of purposive sampling allowed for the selection of participants who are most relevant to our research questions and the targeted approach to recruitment ensured that data collected were appropriate to our study objectives, which has led to more meaningful and focused results. Although the interviews were conducted across the UK in all four devolved nations, study findings may not be generalisable to people outside of these nations. Despite efforts in targeting our study advertisement and the use of a market research company, it proved challenging to recruit participants from a range of ethnic backgrounds and those under the age of 35 for this study.

## Conclusion

This work demonstrates the application of the BCW to help further develop and refine SSS in the UK and help maximize the likelihood of those from LSE groups engaging with smoking cessation support. A lack of knowledge and awareness regarding existing SSS and what they entail was a key barrier to service engagement from LSE groups. Therefore, a co-production approach with community groups and networks helping to disseminate information about SSS and encourage uptake would be beneficial for this population. Further community-based efforts along with a more holistic approach to delivering behavioural support and NRT may prove to be effective in maximizing service reach, engagement, and acceptability. Suggested recommendations based on findings from this study for tailoring UK SSS for LSE groups can be used to inform recommendations for future tobacco control policy including engagement strategies at a local and national level.

## Supplementary Material

Supplementary_Table_1_ntaf256

NTR-2025-391_ntaf256_Supplementary_Table_2-clean_ntaf256_ntaf256

NTR-2025-391_ntaf256_Supplementary_Table_3_ntaf256-clean_ntaf256

## Data Availability

Data sharing is available upon reasonable request. Please contact the corresponding author.

## Abbreviations

NRT, nicotine replacement therapy; BCW, behaviour change wheel; LSE: low socioeconomic; SSS, stop smoking services; TDF: theoretical domains framework
