# Supplementary material for: Enhancing Engagement with Stop Smoking Services among Lower Socioeconomic Groups across the UK: A Qualitative Study using the Behaviour Change Wheel
Source: Nicotine Tob Res. 2025 Dec 19;28(5):857–63. doi: 10.1093/ntr/ntaf256 (PMC13101981; doi:10.1093/ntr/ntaf256)
Supplement: Supplementary_Table_1_ntaf256 [file supplementary_table_1_ntaf256.pdf]

Supplementary Table 1- Definitions of intervention functions

**Intervention functions**	**Definition**
Education	Increasing knowledge or understanding
Persuasion	Using communication to induce positive or negative feelings or stimulate action
Incentivisation	Creating expectation of reward
Coercion	Creating expectation of punishment or cost
Training	Imparting skills
Restriction	Using rules to reduce the opportunity to engage in the target behaviour (or to increase the target behaviour by reducing the opportunity to engage in competing behaviours)
Environmental restructuring	Changing the physical or social context
Modelling	Providing an example for people to aspire to or imitate
Enablement	Increasing means/reducing barriers to increase capability or opportunity[1]

[1] Capability beyond education and training; opportunity beyond environmental restructuring
