# Supplementary material for: Enhancing Engagement with Stop Smoking Services among Lower Socioeconomic Groups across the UK: A Qualitative Study using the Behaviour Change Wheel
Source: Nicotine Tob Res. 2025 Dec 19;28(5):857–63. doi: 10.1093/ntr/ntaf256 (PMC13101981; doi:10.1093/ntr/ntaf256)
Supplement: NTR-2025-391_ntaf256_Supplementary_Table_2-clean_ntaf256_ntaf256 [file ntr-2025-391_ntaf256_supplementary_table_2-clean_ntaf256_ntaf256.docx]

**Supplementary Table 2.** Participant characteristics

|  | | **England** | **Scotland** | **Wales** | **Northern Ireland** | **Total** |
| --- | --- | --- | --- | --- | --- | --- |
|  |  | **n=40** | **n=24** | **n=25** | **n=25** | **n=114** |
| **Smoking status** | Currently smoke | 30  (75%) | 14  (58.5%) | 16  (64%) | 14  (56%) | **74  (65%)** |
|  | Have quit smoking recently (last year) | 5  (12.5%) | 7  (29%) | 6  (24%) | 9  (36%) | **27  (24%)** |
|  | Current e-cigarette user | 5  (12.5%) | 3  (12.5%) | 3  (12%) | 2  (8%) | **13  (11%)** |
| **SSS history** | Accessed a SSS in the last year | 11 (27.5%) | 9  (37.5%) | 5  (20%) | 11  (44%) | **36  (31.5%)** |
|  | Accessed a SSS over one year ago | 18  (45%) | 11  (46%) | 4  (16%) | 11  (44%) | **44  (38.5%)** |
|  | Never accessed a SSS | 11 (27.5%) | 4  (16.5%) | 16  (64%) | 3  (12%) | **34  (30%)** |
| **Gender** | Male | 19  (47.5%) | 12  (50%) | 14  (56%) | 11  (44%) | **56  (49%)** |
|  | Female | 21  (52.5%) | 12  (50%) | 10  (44%) | 14  (56%) | **57  (50%)** |
|  | Non-binary | 0  (0%) | 0  (0%) | 1  (4%) | 0  (0%) | **1  (1%)** |
|  | Prefer not to say | 0  (0%) | 0  (0%) | 0  (0%) | 0  (0%) | **0  (0%)** |
| **Ethnicity** | White British | 32  (80%) | 18  (75%) | 21  (84%) | 23  (92%) | **94  (82%)** |
|  | Asian or Asian British | 5  (12.5%) | 2  (8%) | 1  (4%) | 0 (0%) | **8  (7%)** |
|  | Black or Black British | 1  (2.5%) | 1  (4%) | 0 (0%) | 0 (0%) | **2  (2%)** |
|  | Mixed / Other | 2  (5%) | 3  (12.5%) | 3  (12%) | 2  (8%) | **10  (9%)** |
| **Age** | 18-34 | 12  (30%) | 5  (21%) | 9  (36%) | 8  (32%) | **34  (30%)** |
|  | 35-44 | 12  (30%) | 3  (12.5%) | 4  (16%) | 5  (20%) | **24  (21%)** |
|  | 45-59 | 10  (25%) | 13  (54%) | 10 (44%) | 6  (24%) | **39  (34%)** |
|  | 60 and over | 6  (15%) | 3  (12.5%) | 2  (8%) | 6  (24%) | **17 (15%)** |
| **Individual Indicators of Deprivation** |  |  |  |  |  |  |
| *Area* | Urban | 33  (82.5%) | 22  (92%) | 15  (60%) | 12  (48%) | **82  (72%)** |
|  | Rural | 7  (17.5%) | 2  (8%) | 10  (44%) | 12  (48%) | **32  (28%)** |
| *Education* | Completed a bachelor's degree/masters/PhD | 1  (2.5%) | 3  (12.5%) | 2  (8%) | 1  (4%) | **7  (6%)** |
|  | Completed A levels or equivalent | 5  (12.5%) | 3  (12.5%) | 2  (8%) | 5  (20%) | **15  (13%)** |
|  | O Level or GCSE equivalent | 17  (42.5%) | 6  (25%) | 12  (48%) | 7  (28%) | **42  (37%)** |
|  | Completed further education but not degree | 6  (15%) | 6  (25%) | 3  (12%) | 0  (0%) | **15  (13%)** |
|  | No qualifications/ left school at 16 | 11  (27.5%) | 6  (25%) | 6  (24%) | 12  (48%) | **35  (31%)** |
| *Living Arrangement* | Homeowner (mortgage free) | 2  (5%) | 0  (0%) | 1  (4%) | 0  (0%) | **3  (3%)** |
|  | Homeowner (with mortgage) | 2  (5%) | 0  (0%) | 4  (16%) | 1  (4%) | **7  (6%)** |
|  | Living with family or friends | 7  (17.5%) | 3  (12.5%) | 3  (12%) | 2  (8%) | **15  (13%)** |
|  | Rent from local authority/housing association | 20  (50%) | 17 (71%) | 13  (52%) | 10  (44%) | **60  (53%)** |
|  | Rent privately | 9  (22.5%) | 4  (16.5%) | 4  (16%) | 12  (48%) | **29  (25%)** |
| *Employment Status (highest earner in the household)* | Higher managerial/ professional/ administrator (e.g. Chief executive, senior civil servant, doctor) | 0  (0%) | 1  (4%) | 0  (0%) | 0  (0%) | **1**(1%) |
|  | Intermediate managerial/ professional/ administrative (e.g. middle management, bank manager, teacher) | 1  (2.5%) | 1  (4%) | 0  (0%) | 1  (4%) | **3  (3%)** |
|  | Supervisory/ clerical/ junior managerial/ professional/ administrative (e.g. shop floor supervisor, bank clerk, salesperson) | 5  (12.5%) | 2  (8%) | 1  (4%) | 0  (0%) | **8 (7%)** |
|  | Casual labourer, pensioner, student, unemployed (e.g. pensioner without private pension and anyone living on basic benefits) | 8  (20%) | 4  (16.5%) | 10  (44%) | 4  (16%) | **26  (23%)** |
|  | Skilled manual worker (e.g. electrician, carpenter) | 7  (17.5%) | 9  (37.5%) | 2  (8%) | 1  (4%) | **19  (16%)** |
|  | Semi-skilled and unskilled manual worker (e.g. assembly line worker, refuse collector, messenger) | 8  (20%) | 3  (12.5%) | 6  (24%) | 15  (60%) | **32  (28%)** |
|  | Unemployed | 2  (5%) | 3  (12.5%) | 1 (4%) | 3  (12%) | **9  (8%)** |
|  | Not applicable as there has been no previous employment within the household | 9  (22.5%) | 1  (4%) | 5  (20%) | 1  (4%) | **16  (14%)** |
| **Index of Multiple Deprivation*** | Quintile 1 | 25 (62.5%) | 11(46%) | 11 (44%) | 10 (44%) | **57 (50%)** |
|  | Quintile 2 | 10 (25%) | 4 (16.5%) | 4(16%) | 3 (12%) | **21 (18%)** |
|  | Quintile 3 | 4 (10%) | 4 (16.5%) | 4 (16%) | 2 (8%) | **14 (12%)** |
|  | Quintile 4 | 0 (0%) | 2 (8%) | 1 (4%) | 4 (16%) | **7 (6%)** |
|  | Quintile 5 | 1(2.5%) | 1 (4%) | 5 (20%) | 3 (12%) | **10** (9%) |
|  | Missing | 0 (0%) | 2 (8%) | 0 (0%) | 3 (12%) | **5 (4%)** |
| *Quintile 5 = least deprived, Quintile 1 = most deprived. Deprivation data relate to the IMD 2019, WIMD 2019 or SIMD 2020 deprivation decile. A rank of 1 in all nations = most deprived. Northern Ireland (NI) relates to NIMDM 2017 and cannot be compared to quintiles for England, Scotland, and Wales. | | | | | | |
