# Supplementary material for: Enhancing Engagement with Stop Smoking Services among Lower Socioeconomic Groups across the UK: A Qualitative Study using the Behaviour Change Wheel
Source: Nicotine Tob Res. 2025 Dec 19;28(5):857–63. doi: 10.1093/ntr/ntaf256 (PMC13101981; doi:10.1093/ntr/ntaf256)
Supplement: NTR-2025-391_ntaf256_Supplementary_Table_3_ntaf256-clean_ntaf256 [file ntr-2025-391_ntaf256_supplementary_table_3_ntaf256-clean_ntaf256.docx]

**Supplementary Table 3.** Illustrative quotes aligned to COM-B model and Theoretical Domains Framework

| **COM-B and TDF Mapping** | **Description** | **Illustrative quote** |
| --- | --- | --- |
| Psychological Capability- knowledge | Lack of awareness of service availability, components and processes | "No, to be quite honest with you I wouldn’t even know where to go to find out, I’ve never heard of anything, apart from the leaflet and I heard something where they were sending you vapes to get people off." (TMW008, age 45-59, Wales, currently smoking)  "I knew because I’d been through it before, but the first time I did it, I didn’t know what was going on." (NI3014, age 18-34, Northern Ireland, currently smoking)  “He didn’t realise that you could actually access that stuff free of charge. He thought the same as I think a lot of people do: “I’m not paying £30 for a pack of patches,”" (W3055, age 45-59, Wales, ex-smoker; current e-cigarette user)  "It’s not ignorance, it’s just lack of knowledge that they don’t realise what services are there [...] whereas if they realised they can do it online, they can phone, you don’t actually have to physically go in to the chemist, there is other services out there that can offer support without being face to face and I think that puts people off as well, some people don’t like to do face to face. [...] There’s loads of people there that can actually access them without physically having to leave the house." (TMS3003, age 45-59, Scotland, currently smoking) |
|  | Lack of knowledge surrounding e-cigarettes for cessation | "A better explanation of e-cigarettes and how they can help towards stopping smoking. And again what are the range of the services that would be available, what other products would be available, if you could have an explanation of each item and how it could help you to stop smoking" (NI3015, 60 and over, Northern Ireland, currently smoking) |
| Physical Opportunity- environmental context and resources | Flexible support delivered in a range of modes | “A 10 minute bus journey or no more than half an hour walk. Even if it was a group and I couldn’t make it one day, maybe I’d join online. Options as to whether to go virtually or in person […] I think just openness, if it looks accessible and is accessible” (E3142, age 18-34, England, currently smoking  "I think when you’re rural, unless you can do stuff online or go via your phone, certainly getting around is more of a barrier, because the bus services aren’t great and they so infrequently run and they’re quite expensive whenever you use them as well." (TMNI031, age 45-59, Northern Ireland, used to smoke)  "It was all done via the telephone. I don't think they offer an in person support service anymore [...] I did go to a group session years ago and I did find it helpful" (S3003, age 45-59, Scotland, currently smoking) |
|  | Continued/frequent behavioural support | "Even if it was like a phone call, like a text, or even an email or something. Sending someone just a message and go, “How are things going for you?” [...] Because most of the time if you do something like that, as soon as you’ve give up, the person will go, “Right, my job’s done” but six months down the line that person could be smoking again." (W3005, age 18-34, Wales, currently smoking)  "I could contact them at any time either via WhatsApp [...] if I was having days where I was struggling, she says, “Just phone me [...] My phone’s always on until 6 o’clock" [...]it was good that way, it was good" (S3009, age 45-59, Scotland, used to smoke)  "It worked very well for the first six weeks but after that the support was a bit erratic, you never know when you're going to get a phone call or not." (S3022, 60 and over, Scotland, currently smoking) |
|  | Community-based support | "It’s always nice when you go to a place that you're familiar with, which I was lucky, my clinic is 10 minutes away from me." (S3022, 60 and over, Scotland, currently smoking)  "I think also they mentioned that there might be in-person groups, but I think they were mostly out the city in areas where I didn’t want to go. Just because of my own conditions travelling can be a bit tricky." (TMS022, age 45-59, Scotland, used to smoke) |
|  | Lack of time to access SSS | "You could attend meetings or you had phone calls but because I had a busy lifestyle I never bothered with it." (W3011, age 35-44, Wales, currently smoking) |
|  | Lack of behavioural support in pharmacies | " I don’t want to be mean to [pharmacy] there but there was no actual support other than, “Into this room, blow in this device, there you go, see you next week.” You were literally, you were in and out quicker than you can go to the doctor sometimes today" (W3055, age 45-59, Wales, used to smoke; current e-cigarette user)  “Unfortunately where I stay in [Scottish city] the pharmacy is really busy, so they’ve not got the time, they just give you the patches and tell you, “Good luck”” **(S3019, age 45-59, Scotland,** currently smoking**)**  "I know various people that have tried to stop smoking but basically said the same as me, there is no real back-up and they always felt that they only had a limited time to discuss with the pharmacist." (NI3033, age 60 and over, Northern Ireland, currently smoking) |
|  | Free NRT support | "They’re so expensive too. That wee inhaler and the cartridge is about £20 a hit. And then you’re talking about the patches on top of that [...] I thought it was a good service that they provide, absolutely, 100%." (TMNI013, age 45-59, Northern Ireland, used to smoke)  "We get free prescriptions here in Scotland, so it’s a free service, which is very good actually" (S3003, age 45-59, Scotland, currently smoking)  “I probably couldn’t afford do it myself if I was paying for it**”** (**S3018, age 60 and over, Scotland,** currently smoking**).​** |
|  | Difficulty in accessing NRT | “I wanted a different flavour, and she sent me the one I didn’t like out again. I thought, “This is useless” (E3123, age 60 and over, England, used to smoke)  "I’d come home with the prescription and then I’d give it to my husband to go and get it down the other chemist. The road was horrendous to cross, so I used to think, “Oh, I’ll just go home with it,” but I think accessibility needs to be an issue." (W3049, age 45-59, Wales, currently smoking) |
|  | Lack of choice for in-person support | "It was all done via the telephone. I don't think they offer an in-person support service anymore [...] I did go to a group session years ago and I did find it helpful because there was the peer group and there was a wee bit of encouragement from fellow smokers to stop smoking. That was good" (S3003, age 45-59, Scotland, currently smoking) |
| Social Opportunity- social influences | Social support | "I must just say my daughter, my son and my partner never even noticed, even a week after [...] I was absolutely gutted, they never noticed. " (TMW032, age 60 and over, Wales, used to smoke) |
|  | Social networks and the use of a peer support model to support quitting | " I think doing it with someone is easier for me because you're both doing it and it’s not say like you and your partner, your partner is next to you smoking and you’re trying to give up." (TMW030, age 18-34, Wales, used to smoke)  "I think it would be beneficial to maybe be able to speak to other smokers and people that have quit [...] I think it would be very motivating [...] being able to speak to other people not just health experts but actual people that have smoked and have stopped smoking, I think being able to have a conversation with them would be very beneficial, I think it would motivate you a lot to try to quit." (NI3029, age 18-34, Northern Ireland, currently smoking)  “I did go to a group session years ago and I did find it helpful because there was the peer group and there was a wee bit of encouragement from fellow smokers to stop smoking. That was good" (S3003, age 45-59, Scotland, currently smoking) |
|  | Rapport with advisor | "I think the only way to make it better it just to try and keep it as friendly as possible and try and try and keep it as informal as possible in a way. More as like a friend-to-friend basis than a professional basis." (W3060, age 18-34, Wales, used to smoke; current e-cigarette user)  "I think it’s very good, I think flexibility’s important. Friendliness is there, giving good advice, and when I have only been able to stop for a couple of days, you know, they’ve been supportive and encouraged me to keep on at it. And it’s non-judgemental" (NI3013, 60 and over, Northern Ireland, currently smoking) |
|  | Smoking related stigma (fear of failing and wanting to quit on their own) | “I think you're treated as a leper sometimes”. (E3067, age 45-59, England, currently smoking)  "you just feel that you don’t belong, because I’ve always been surrounded by smokers through work and friends [...] so there was a bit of peer pressure as well [to successfully quit]" (S3027, age 35-44, Scotland, used to smoke)  "Because it is, it’s classed as an antisocial habit now [...] whereas years ago it was socially accepted, now it’s not really, it’s frowned upon, you feel like an outcast when you smoke" (TMS3003, age 45-59, Scotland, currently smoking) |
| Reflective Motivation- beliefs about capabilities and beliefs about consequences | Negative beliefs about the efficacy of behavioural support | " I wasn’t really after talking to someone for hours and hours about it, that was the main thing that put me off." (W3046, age 45-59, Wales, used to smoke)  “Discussing my, although they’re personal habits, they’re not exactly private but it’s just not something that I would feel comfortable sitting down with people and discussing” **(S3050, age 45-59, Scotland, used to smoke; current e-cigarette user). ​**  “I decided not to go because I don’t really like people and if I haven’t got any nicotine in my system, … I just don’t want to snap at people I would rather just stay at home” (E3078, age 18-34, England, currently smoking)  "I imagine these are self-help groups that would be constantly discussing it and bringing it to the forefront whereas I would rather forget it and not think about it. The less I think about it, the easier it would be for me”. (TMNI024, age 35-44, Northern Ireland, used to smoke) |
|  | Negative beliefs about NRT efficacy | “The thing that worries me is if I'm ever told take this Nicorette gum instead and I’ve heard such horror stories” (E3019, age 45-59, England, currently smoking) |
